# Seven‐day vonoprazan‐based triple therapy as first‐line *Helicobacter pylori* treatment in comparison with extended sequential therapy

**DOI:** 10.1002/jgh3.12858

**Published:** 2023-01-11

**Authors:** Yu‐Tse Chiu, Fu‐Jen Lee, Chen‐Ya Kuo, Yang‐Chao Lin, Kai‐Shun Liang, Liang‐Wei Tseng, Yu‐Tsung Chen, Chi‐Yang Chang

**Affiliations:** ^1^ Division of Gastroenterology and Hepatology, Department of Internal Medicine Fu Jen Catholic University Hospital New Taipei City Taiwan; ^2^ School of Medicine, College of Medicine Fu Jen Catholic University New Taipei City Taiwan

**Keywords:** antibacterial agents, *Helicobacter pylori*, potassium‐competitive acid blocker, proton pump inhibitors

## Abstract

**Background and Aim:**

Vonoprazan as a new acid blocker has more potency and longer lasting acid suppression than proton pump inhibitors. Whether the efficacy of vonoprazan‐based triple therapy is comparable with or even better than that of currently recommended first‐line therapies is still unknown. Our study aims to compare the eradication rate and major adverse effects between 7‐day vonoprazan‐based triple therapy with high‐dose amoxicillin and 14‐day extended sequential therapy.

**Methods:**

We performed a retrospective analysis from the database of ^13^C‐urea breath test at Fu Jen Catholic University Hospital. All patients with a definite diagnosis of *Helicobacter pylori* infection by rapid urease test, urea breath test, stool antigen test, or pathology report were recruited. Patients receiving first‐line regimens with vonoprazan‐based triple therapy or extended sequential therapy were included. The respective eradication rate determined by ^13^C‐urea breath test and major adverse effects were demonstrated.

**Results:**

Totally, 106 patients were recruited in the vonoprazan‐based triple therapy group and 357 in the extended sequential therapy group. There was no significant difference in eradication rate between vonoprazan‐based triple therapy with high‐dose amoxicillin and extended sequential therapy (83.0 vs 88.8%, *P* = 0.12). Major adverse effects occurred in 13 of the extended sequential therapy group but none in the other group (0% vs 3.6%, *P* = 0.046).

**Conclusions:**

Seven‐day vonoprazan‐based triple therapy with high‐dose amoxicillin is a potential first‐line anti‐*Helicobacter pylori* regimen alternative to current standard treatment, with the advantages of simplicity, short treatment duration, low pill burden, and fewer major adverse effects.

## Graphical abstract

Seven‐day vonoprazan‐based triple therapy with high‐dose amoxicillin is a potential first‐line anti‐*Helicobacter pylori* regimen alternative to current standard treatment, with simplicity, short treatment duration, low pill burden, and fewer major adverse effects.

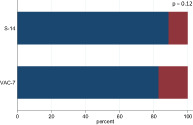

## Introduction

*Helicobacter pylori* (*H. pylori*) is a well‐established risk factor for peptic ulcer disease and gastric cancer.^1^ A global statistics taken in 2020 ranked gastric cancer as the sixth most common cancer worldwide and the third leading cause of cancer death.^2^ It is estimated that over 85% of non‐cardia gastric cancer is attributable to *H. pylori*,^3^ meaning that the majority of gastric cancer can be prevented through *H. pylori* eradication. The extended sequential therapy provides a satisfactory eradication rate of up to 90.7% and can replace conventional triple therapy in the era of increasing antimicrobial susceptibility.^4^ However, the complexity of its “sequential” usage along with the need of changing antibiotic drugs during the treatment course is sometimes confusing to the patients.^5^ A novel class of acid‐suppressing agents, the potassium‐competitive acid blocker (P‐CAB), has emerged in recent years. Vonoprazan as a kind of P‐CAB has a more potent and longer lasting acid suppression effect than conventional proton pump inhibitors (PPIs), which had been proven to be vital in *H. pylori* eradication.^6^ As a result, vonoprazan‐based eradication therapy is expected to have better efficacy with a shorter treatment duration.^7^ One meta‐analysis, which included 10 case–control trials, showed that 7‐day vonoprazan‐based triple therapy had a significantly higher eradication rate than conventional 7‐day PPI‐based triple therapy (87.9% vs 72.8%, by intention‐to‐treat analysis).^8^ However, PPI‐based triple therapy should no longer be recommended as the first‐line treatment in ares with high clarithromycin resistance.^5^ Therefore, there is still insufficient evidence supporting the first‐line use of vonoprazan‐based triple therapy. Besides, given that the recommended dose of amoxicillin in the conventional PPI‐based triple therapy is 1000 mg b.i.d. rather than 750 mg b.i.d., which is a suboptimal dose in the trials included in the above meta‐analysis,^8^, ^9^ it is hypothesized that titration of amoxicillin to 1000 mg b.i.d. may further increase the efficacy of the vonoprazan‐based regimen. Vonoprazan‐based triple therapy with high‐dose amoxicillin (1000 mg b.i.d.) has been prescribed since April 2021 in Fu Jen Catholic University hospital. Meanwhile, the 14‐day extended sequential therapy has been recommended by guidelines as first‐line anti‐*H. pylori* therapy^9^ and widely adopted in our hospital. This study aimed to compare the efficacy of the extended sequential therapy (abbreviated as S‐14 below) with that of vonoprazan‐based triple therapy with high‐dose amoxicillin (abbreviated as VAC‐7 below) in a retrospective way.

## Methods

### Subjects

We performed a retrospective analysis from the database of ^13^C‐urea breath test (UBT) in Fu Jen Catholic University Hospital from 1 April 2021 to 31 December 2021. Patients were deemed to have *H. pylori* infection through a positive rapid urease test, *H. pylori* stool antigen test, urea breath test (UBT), or pathology report showing *H. pylori* in tissues. Those with only positive anti‐*H. pylori* IgG or those referred from other medical facilities for *H. pylori* eradication without records of the diagnostic modalities were excluded. Patients with *H. pylori* infection and receiving first‐line therapy were recruited and divided into two groups according to their anti‐*H. pylori* regimen: the VAC‐7 group (vonoprazan 20 mg + amoxicillin 1000 mg + clarithromycin 500 mg twice daily for 7 days) and the S‐14 group (PPI [lansoprazole 30 mg/rabeprazole 20 mg/pantoprazole 40 mg] + amoxicillin 1000 mg twice daily for 7 days, followed by the same PPI + clarithromycin 500 mg + metronidazole 500 mg twice daily for 7 days).

### Outcomes

The primary endpoint is the eradication rate of *H.pylori* (evaluated by UBT at least 4 weeks after completion of the therapy) on an intention‐to‐treat basis; the secondary endpoint is the rate of major adverse events (AEs). The criteria for major AEs were based on the Common Terminology Criteria for Adverse Events (CTCAE) v5.0,^10^ and any subjective discomfort occurring during the treatment, which corresponds to AEs of second grade in CTCAE v5.0 (symptomatic; limiting instrumental ADL) or above, or causes earlier return to the clinic or discontinuation of the therapy, was deemed as a major adverse effect.

### Statistical analysis

Basic characteristics, eradication rate, and major AEs were compared between the VAC‐7 group and the S‐14 group using StataSE 14 by Wilcoxon rank sum test or Chi‐squared test as indicated, and a *P*‐value less than 0.05 was deemed as statistically significant. In case of nonsignificant results regarding the eradication rate, a non‐inferior test by the Farrington–Manning score test using SAS OnDemand for Academics was performed in addition. Furthermore, subsequent regimens for treatment failure cases were also analyzed, and the eradication rates were compared between groups, based on either their original first‐line regimen or the subsequent second‐line regimen, by the Chi‐squared test.

### Ethical consideration

Our study was approved by the institutional ethics committee of our hospital (FJUH‐IRB number: FJUH111225). There was no financial support for this study.

## Results

**Figure 1:**
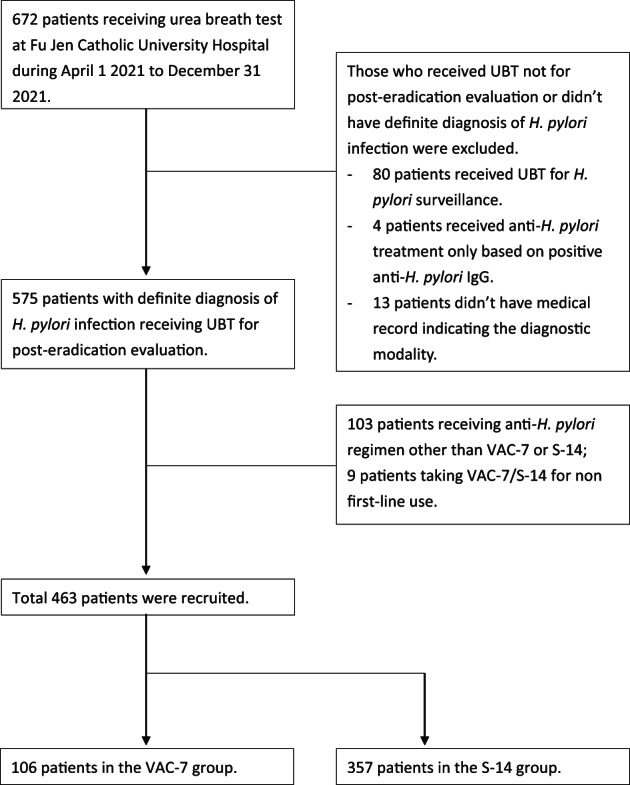
Flowchart of patient recruitment.

**Figure 2:**
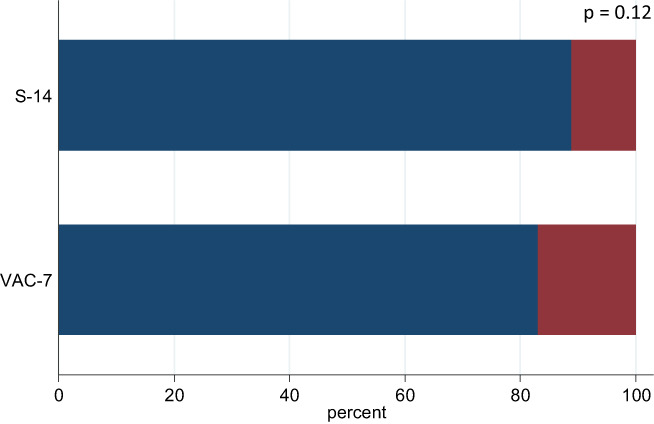
Proportional stacked bar graph of the eradication rate among the two groups. 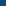 Treatment success; 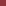 Treatment failure.

A total of 672 patients underwent UBT at Fu Jen Catholic University Hospital from 1 April 2021 to 31 December 2021 (Fig. 1). Those who underwent UBT not for post‐eradication evaluation, or did not have a definite diagnosis of *H. pylori* infection, were excluded. Nine patients received VAC‐7/S‐14 for second‐line therapy (six in the VAC‐7 group, five of whom failed the eradication; three in the S‐14 group, one of whom failed) and were thus excluded. Finally, 106 patients receiving VAC‐7 and 357 patients receiving S‐14 were recruited. The basic characteristics of patients are listed in Table 1. There was no significant difference in eradication rate between VAC‐7 and S‐14 (83.0% *vs* 88.8%, *P* = 0.12; Table 2 and Fig. 2).

**Table 1:** Basic characteristics

Anti‐*Helicobacter pylori* regimen	VAC‐7 (*n* = 106)	S‐14 (*n* = 357)	*P*‐value
Basic characteristics			
Age (years), mean ± SD	55.4 ± 13.2	54.4 ± 13.0	0.55
Male, *n* (%)	41 (38.7)	201 (56.3)	<0.01
Diagnostic tools			<0.01^†^
Rapid urease test, *n* (%)	72 (67.9)	214 (59.9)	
Pathology of endoscopic biopsy, *n* (%)	6 (5.7)	82 (23.0)	
Both positive for rapid urease test and pathology of endoscopic biopsy, *n* (%)	1 (0.9)	18 (5.0)	
Invasive ways, *n* (%)	79 (74.5)	314 (88.0)	
Stool antigen test, *n* (%)	15 (14.2)	25 (7.0)	
Urea breath test, *n* (%)	12 (11.3)	18 (5.0)	
Noninvasive ways, *n* (%)	27 (25.5)	43 (12.0)	
Endoscopic findings with active ulcers, *n* (%)			
Active gastric/duodenal ulcers, *n* (%)	1 (0.9)	197 (55.2)	<0.01
Active gastric ulcers, *n* (%)	0 (0)	132 (37.0)	<0.01
Active duodenal ulcers, *n* (%)	1 (0.9)	109 (30.5)	< 0.01

^†^ Since there was overlap between the diagnostic modalities (some were both positive for rapid urease test and pathology of endoscopic biopsy), we compared the ratio of invasive ways (rapid urease test or pathology of endoscopic biopsy; *n* = 79/314) and noninvasive ways (urea breath test or stool antigen test; *n* = 27/43) between the two groups using Chi‐square test, which showed significant difference (*P* < 0.01).

**Table 2:** Results

*Helicobacter pylori* eradication regimen	VAC‐7 (*n* = 106)	S‐14 (*n* = 357)	*P*‐value
Eradication rate, *n* (%)	88 (83.0)	317 (88.8)	0.12
Major adverse effects, *n* (%)	0 (0)	13 (3.6)	0.046

Subsequent treatment for treatment‐failure cases				
The second‐line regimen		VAC‐7 (*n* = 18)	S‐14 (*n* = 40)	*P*‐value^†^
Levofloxacin‐based therapy, *n* (%)	Total	4	14	0.89
	Eradication rate, *n* (%)	3 (75)	10 (71.4)	
Bismuth‐based quadruple therapy, *n* (%)	Total	13	23	0.45
	Eradication rate, *n* (%)	9 (69.2)	13 (56.5)	
*P*‐value for the comparison between Levofloxacin‐ based therapy and Bi‐based quadruple therapy		0.83	0.37	‐
No further treatment, *n* (%)		1 (5.6)	3 (7.5)	‐

^†^ *P*‐value for the comparison of the same second‐line regimen between VAC‐7 group and S‐14 group.

The non‐inferior test by the Farrington–Manning score test with a margin of 0.10 showed no significance between the two groups (*P* = 0.16). Major AEs occurred in 13 of the S‐14 group while none in the other group (*P* = 0.046; Table 2). AEs included diarrhea (*n* = 2), vomiting (*n* = 2), skin rashes (*n* = 3), abdominal pain (*n* = 3), and general malaise (*n* = 1). There was no detailed description of the AEs for the remaining two cases. Four of them did not complete the regimen, and five failed *H. pylori* eradication. Those who failed first‐line treatment received second‐line therapy based on the clinician's judgment, except for four who declined further treatment (Table 2). The rest took levofloxacin‐based therapy [either levofloxacin triple therapy (PPI + amoxicillin 1000 mg + levofloxacin 250 mg twice daily for 10–14 days) or levofloxacin sequential therapy (PPI + amoxicillin 1000 mg twice daily for 7 days, followed by the same PPI + levofloxacin 250 mg + metronidazole 500 mg twice daily for 7 days)] or bismuth‐based quadruple therapy (PPI twice daily, with bismuth 324 mg + tetracycline 500 mg four times a day and metronidazole 500 mg thrice daily, for 10–14 days). The eradication rates of the second‐line treatment, evaluated by UBT using intention‐to‐treat analysis, were all around 70–75% except that of bismuth‐based quadruple therapy in the S‐14 group (only 56.5%). Patients receiving levofloxacin‐based therapies and originally belonging to VAC‐7 group tended to have a higher eradication rate, although there was no statistical significance.

## Discussion

Vonoprazan as a novel acid blocker prevails over conventional PPIs in terms of potency, duration, and stability of acid suppression thanks to its feature of accumulation in parietal cells and hence slower clearance from gastric glands. Its effectiveness is unaffected by ambient pH and its metabolism is independent of CYP2C19.^11^, ^12^, ^13^ Based on above facts, vonoprazan was expected to be at least as effective as PPIs as part of anti‐*H. pylori* regimens. The first study of vonoprazan‐based triple therapy appeared in 2016 as a Phase III trial with excellent results.^14^ Several studies came out afterward in support of the use of vonoprazan‐based triple therapy as one of the first‐line anti‐*H. pylori* regimens.^8^ However, through retrospective analysis, we found that the eradication rate of VAC‐7 was not as high as reported in the literature but was still comparable with that of S‐14 (83.0% vs 88.8%, *P* = 0.12). Since there is one study reporting a lower eradication rate of vonoprazan‐based triple therapy with low‐dose amoxicillin in strains resistant to clarithromycin (76.2%),^15^ we postulated that the strain with increased resistance endemically might contribute to the below‐expected eradication rate.

Although it had been reported that some other vonoprazan‐containing regimens show effectiveness for second/third‐line anti‐*H. pylori* therapy,^14^, ^16^, ^17^ VAC‐7 seemed not to be a good choice. Six participants in the VAC‐7 group took other anti‐*H. pylori* regimens before, and five of them failed the eradication. Although the sample size is small, this suggested that we should avoid using VAC‐7 as the second‐line anti‐*H. pylori* therapy.

Levofloxacin‐based triple therapy and bismuth‐based quadruple therapy could serve as salvage treatment after primary therapy fails,^9^ and levofloxacin‐based sequential therapy was also proposed to be a potent second‐line regimen, which was found to be even more effective than levofloxacin‐based triple therapy.^18^ These regimens are the most commonly used second‐line therapies in our hospital. Their eradication rates all fell below 80% in this study, probably due to the local resistant strains, the intention‐to‐treat analysis, and the small sample size. Overall, these salvage regimens did not underperform in the VAC‐7 group; instead, the bismuth‐based quadruple therapy even tended to do better than in the S‐14 group, although no significance was achieved. This fact provided us the clue that the strategy of selecting a salvage regimen after VAC‐7 may be not too different from that after S‐14. However, owing to the limitation of our small sample size, the optimal salvage treatment after VAC‐7 needs more investigation.

The advantage of our study is that we compared the vonoprazan‐based triple therapy with the currently recommended first‐line anti‐*H. pylori* regimens (including the extended sequential, concomitant, or hybrid therapy) by clinical guidelines.^9^ However, there are some shortcomings secondary to its retrospective nature. The first one is the unbalanced distributions regarding the basic characteristics. Reviewing the literature, other than anti‐*H. pylori* regimen, the eradication rate is positively correlated with advanced age and inversely associated with the presence of a gastric ulcer and poor medication compliance.^19^, ^20^, ^21^ Since vonoprazan, although already approved by the Taiwan Food and Drug Administration, has not been reimbursed by Taiwan National Health Insurance (TNHI) yet and only patients with peptic ulcers can take anti‐*H. pylori* therapy reimbursed by TNHI, we prescribed VAC‐7 mainly to those without peptic ulcers, which is why more patients in the S‐14 group were diagnosed to have *H. pylori* infection through invasive ways (88.0% vs 74.5%, *P* < 0.01) and presented with gastric ulcers (36.9% vs 2.7%, *P* < 0.01). Second, without TNHI reimbursement the VAC‐7 was prescribed less frequently than S‐14, causing an unbalance of the patient number and inadequate power for a non‐inferior test. Third, because not all minor AEs were recorded in the medical chart, we could analyze the occurrence of only the major ones. Similarly, compliance was hard to assess retrospectively and we could compare the eradication rate only by intention‐to‐treat analysis. Last but not least important, data regarding antibiotic resistance are not available also because of the retrospective nature.

## Conclusion

Seven‐day vonoprazan‐based triple therapy with high‐dose amoxicillin is a potential first‐line anti‐*H. pylori* regimen alternative to current standard treatment. It has the advantages of simplicity, short treatment duration, low pill burden, and fewer major AEs. However, whether its eradication rate is comparable with that of the extended PPI‐based sequential therapy requires further investigation by a large‐scale randomized control trial in the future.

## Ethics statement

This study was approved by the Institutional Ethics Committee of our hospital (FJUH‐IRB number: FJUH111225).
